# Editorial: Community series in tools, techniques, and strategies for teaching in a real-world context with microbiology, volume II

**DOI:** 10.3389/fmicb.2023.1156805

**Published:** 2023-02-21

**Authors:** Davida S. Smyth, Nichole A. Broderick, Carlos C. Goller

**Affiliations:** ^1^Department of Life Sciences, College of Arts and Sciences, Texas A&M University-San Antonio, San Antonio, TX, United States; ^2^Johns Hopkins University, Baltimore, MD, United States; ^3^Biotechnology Program (BIT), North Carolina State University, Raleigh, NC, United States

**Keywords:** guided inquiry, scientific literacy, course-based undergraduate research experience (CURE), underrepresented minority (URM) students, diversity

This Research Topic is the second volume in the Community Series Tools, Techniques, and Strategies for Teaching in a Real-World Context with Microbiology. Making microbiology relevant to our students increases student engagement with science, which could not be more important as we emerge from the COVID-19 pandemic. We have learned much during this period as we adapted and modified our learning environments and strategies, all while demonstrating the many ways microbes impact our world beyond disease. Placing microbes at center stage leads to engaging and exciting curricula and assignments. Microbes also are wonderful tools as they are easy to manipulate in the laboratory and serve as model organisms helping us to study biological phenomena and concepts. As faculty we can engage our students through guided inquiry, tactile hands-on tools, authentic research experiences, project-based learning, and case studies, with valuable, student-centered experiences occurring both inside and outside the classroom. We can also endeavor to abide by the principles of equity, diversity, and inclusion and those of universal design allowing all students to succeed in our courses.

Of the five submissions, three focused on course-based research experiences (CUREs), a strategy known to promote student engagement with science while reducing barriers to participation in research. The first by Tarín-Pelló et al. examined the impact of the SWICEU antibiotic discovery project, on student knowledge of antimicrobial resistance (AMR) and the use of antimicrobials over 5 years in a population of pre-university students in a region of Spain, the country with the fifth highest consumption of antibiotics in Europe. The authors had the goal of using education to promote social awareness of the problem of AMR. Using a multiple-choice question survey, pre- and post-participation along with a Likert-scale- satisfaction survey they showed that student knowledge of the problem of AMR increased. This work is significant not only in scale but also in the nature of the student population targeted, being pre-university students. In the study of Sun et al. the authors leveraged existing datasets to develop an online data science CURE in microbiology, to ensure that students could participate in research at the onset of the COVID-19 pandemic. Students worked with large publicly-available datasets and developed and executed a research project, the results of which were disseminated in the online Undergraduate *Journal of Experimental Microbiology and Immunology*. Notably, the study utilized peer review by subject matter experts to demonstrate success. The last submission by Roberts and Shell developed an opportunity for students to participate in research through a molecular biology course-based laboratory research experience. It was an authentic, guided research experience with the research questions coming directly from one of the faculty member's labs, focusing on post-transcriptional regulation in mycobacteria. The authors developed a customized “Skills and Concepts Inventory” survey to gauge student learning. These three submissions highlight the range of ways that a CURE-based curriculum can be adopted, even during a pandemic, and designed to withstand challenges and barriers to implementation, such as time management and organization, mode of delivery, balancing depth and breadth, and ensuring authenticity. CUREs are a valuable and proven way to engage students in the research process and to help grow their science capital and sense of belonging in STEM. In addition, students in these courses become not only participants but contributors to the research, protocols, and materials of the faculty member's lab.

The COVID-19 pandemic demonstrated the need for a scientifically literate populace. In their perspectives piece, Harris et al. tackled the pressing issue of scientific literacy. The public's distrust and misunderstandings of science related to vaccines demonstrate the critical need of ensuring a broad understanding of microbial-human interactions, how our immune system responds to infection, and how vaccines are developed, produced, and delivered. While many of us teach these topics to our students in undergraduate and graduate-level courses, these concepts are critically important to broader audiences, including younger students and the general public. Harris et al. leveraged the “Tactile Teaching Tools with Guided Inquiry Learning (TTT-GIL)” method^1^, to develop an interactive molecular puzzle, based on the lac operon. They report on two TTT-GIL activities that explore molecular interactions of the immune system and are tailored to diverse learners ranging from middle schoolers to master's students. The activities built upon the valuable Process-Oriented Guided Inquiry Learning (POGIL) method^2^ and the learning cycle to enable students to construct new concepts. Of note is their use of Universal Design for Learning (UDL) principles supporting the learning of deaf and hard-of-hearing middle and high school students. These activities will allow any learner to explore the fundamentals of immunology and be broadly adaptable to a variety of learning contexts. Additional information on Universal Design for Learning principles can be found on the QUBES Hub^3^.

Our last submission also used a guided inquiry (TTT-GI) activity, where students in two sections of a large enrollment Human Anatomy and Physiology course at a research-intensive (R1) university explored how the gut microbiome fermented carbohydrates, helping them to understand the role of the gut microbiome and its physiological impacts on human and animal health. Delivered in a hyflex format, students attending in person used commercially available toys to assemble structurally complex, representative carbohydrates, while online students used a digital learning tool to construct the carbohydrates in a virtual forum. Students made predictions about how these structures would be fermented into short-chain fatty acids (SCFAs) and what role these SCFAs would play physiologically. Using pre-and post-assessment and three research questions, results were favorable and the learning objectives were attained. The delivery format was also assessed revealing that while the delivery method had little impact on learning, the two sections differed in impact, likely due to differences between the length and spacing of the activity. For diverse students traditionally underrepresented in STEM (first-generation students and students with one or more disabilities) the authors noted modest and non-significant positive learning gains for some populations, but that students who had already completed upper-level biology courses exhibited the greatest learning gains. This activity is flexible and adaptable to any anatomy and physiology course and would work in any number of course formats.

We hope that you find these articles of use and value and that you consider some of the approaches described for your own classrooms. It is important to recognize lessons learned as we delivered our courses in a variety of formats and in different institutional contexts. We valued our colleague's consideration of diverse students, those traditionally underrepresented in STEM, and the use of universal design principles. Guided and scaffolded approaches helped all students achieve the desired learning outcomes. Course-based research allowed all students to participate and engage in valuable research experiences. We are grateful to our colleagues for their contributions, particularly as we struggled through the COVID-19 pandemic, and hope that you will continue to share your ideas and resources that we can improve educational opportunities for all students, develop the next generation of researchers and promote scientific literacy and understanding of the process of science for all learners.

## Author contributions

DS, NB, and CG wrote and edited the text. All authors contributed to the article and approved the submitted version.

## Footnotes

^1^ https://qubeshub.org/community/groups/stembuild/overview

^2^ https://pogil.org/

^3^ https://qubeshub.org/community/groups/udl

